# On the role of eye contact in gaze cueing

**DOI:** 10.1038/s41598-018-36136-2

**Published:** 2018-12-14

**Authors:** Kyveli Kompatsiari, Francesca Ciardo, Vadim Tikhanoff, Giorgio Metta, Agnieszka Wykowska

**Affiliations:** 1 Istituto Italiano di Tecnologia, Social Cognition in Human-Robot Interaction, via Enrico Melen 83, 16152 Genova, Italy; 2 Ludwig Maximilian University, Großhaderner Str. 2, 82152 Planegg, Germany; 3 Istituto Italiano di Tecnologia, iCub Facility, Via Morego 30, 16163 Genova, Italy; 4 University of Plymouth, Drake Circus, PL4 8AA Plymouth, UK

## Abstract

Most experimental protocols examining joint attention with the gaze cueing paradigm are “observational” and “offline”, thereby not involving social interaction. We examined whether within a naturalistic online interaction, real-time eye contact influences the gaze cueing effect (GCE). We embedded gaze cueing in an interactive protocol with the iCub humanoid robot. This has the advantage of ecological validity combined with excellent experimental control. Critically, before averting the gaze, iCub either established eye contact or not, a manipulation enabled by an algorithm detecting position of the human eyes. For non-predictive gaze cueing procedure (Experiment 1), only the eye contact condition elicited GCE, while for counter-predictive procedure (Experiment 2), only the condition with no eye contact induced GCE. These results reveal an interactive effect of strategic (gaze validity) and social (eye contact) top-down components on the reflexive orienting of attention induced by gaze cues. More generally, we propose that naturalistic protocols with an embodied presence of an agent can cast a new light on mechanisms of social cognition.

## Introduction

Joint attention (JA) is an important mechanism of non-verbal communication for social interactions. JA occurs when two or more individuals direct their attention to the same event or object in the environment^1^ and it can be induced by directional (social) gestures, such as gaze shifts. In order to experimentally investigate gaze-related mechanism of JA, variations of the Posner paradigm^2^ have been developed and extensively employed^3–5^. In such paradigms, a face (often schematic) is typically presented centrally on a screen, first with gaze straight-ahead, and then with gaze averted towards a lateral location on the screen. Subsequently, a target typically appears either at the location where the gaze was directed (validly cued target), or at a different location (invalidly cued target). Response times (RTs) in target detection or discrimination are typically faster for validly cued targets compared to invalidly cued targets, reflecting the *gaze-cueing effect* (GCE). The GCE is observed even when the gaze is counter-predictive (i.e. the target is more likely to appear in the invalidly cued locations), indicating that directional gaze elicits a reflexive attentional shift towards the gazed-at location^3–5^. However, recent studies suggest that gaze following can be top-down modulated through task demands and goals, inferred goals of the observed agent, or beliefs about his/her agency^6–12^.

Despite the limited amount of gaze cueing studies using another human as a central cue^13,14^, the majority of studies examine JA through *offline* protocols with social stimuli presented statically on a screen^15^. However, screen-based offline paradigms lack ecological validity, and they might fail to capture true social cognitive mechanisms evoked in natural social interactions^15^. One of crucial mechanisms is real-time eye contact^16^, which informs about readiness to engage in interaction. Eye contact has been shown to affect various cognitive processes and states, like attention, memory, and arousal^17–28^. For instance, Senju and Hasegawa^20^ presented a face on a screen with different gaze directions (direct, averted, closed eyes) followed by a peripheral target^20^. RTs were slower for direct gaze compared to averted gaze or closed eyes, suggesting that eye contact delayed attentional disengagement from the face. Similarly, Bristow *et al*.^21^ measured behavioral and neural responses to gaze shifts directed (or not) to a target as a function of the social context (social: eye contact, non-social: averted gaze) and the goal-directedness of the gaze shift (i.e. toward the target or not). Authors found that an eye contact preceding the gaze shift facilitated gaze shift detection, suggesting that participants’ attention was covertly attracted to the social context of the face. Moreover, authors reported greater activation in the medial prefrontal cortex and precuneus with respect to goal-directed and social gaze shift compared to non-goal-directed and non-social shift, suggesting that this activity may reflect the experience of JA associated with these gaze shifts^21^. More recent studies investigated the effect of eye contact on attentional processes, by employing measures of either oculomotor behavior for screen-based paradigms^22–24^ or even during real-time social interactions^25^. In a series of screen-based studies, Dalmaso *et al*.^22,23^ reported that eye contact can modulate spatial and temporal parameters of goal-directed saccades (i.e., greater saccadic curvature, decreased peak velocity). Finally, a very recent study by Xu and colleagues^26^ revealed a larger GCE following a supraliminally presented direct gaze in comparison to gaze directed downwards.

Previous studies have found that real-time direct gaze enhances EEG asymmetry (i.e., less power alpha band from left-sided frontal channels) and skin conductance responses (an index of arousal) compared to a direct gaze presented on a screen^27,28^. Additionally, Hietanen *et al*.^25^ found that a real-time eye contact can shape attentional mechanisms differently than pictures^20,24^. Hietanen *et al*.^25^ reported that real-time eye contact with a confederate enhanced performance (i.e. faster responses) in both discrimination and Stroop tasks. The authors^25^ proposed that real-time eye contact might have increased autonomic activation. Therefore, it is plausible to assume that real-time mutual gaze embedded in a gaze-cueing paradigm might affect the processing of socially relevant sensory information, thereby modulating JA effects.

**Figure 1:**
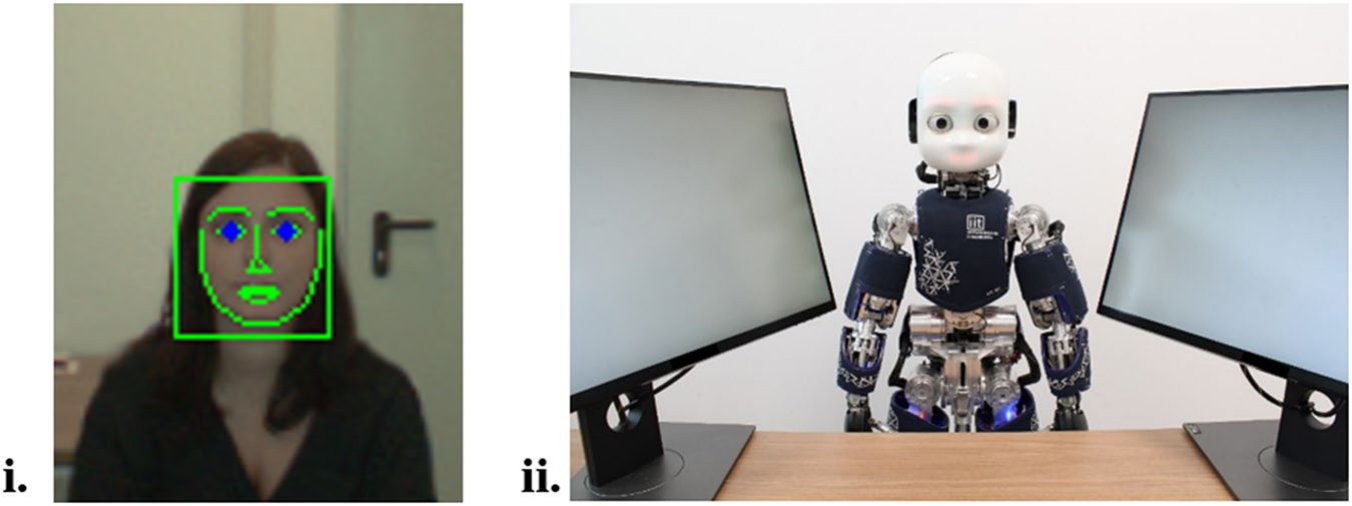
Panel i.: Example of the output of the face detector algorithm drawn from the left robot eye. Panel ii.: Experimental setup from participant’s point of view.

Recent approaches to the study of the mechanisms of social cognition propose that more interactive experimental protocols are crucial for understanding cognitive and social mechanisms elicited by social interaction^29–33^. In line with this approach, we used a novel method of involving an embodied humanoid robot in an online interactive experimental manipulation. More specifically, we embedded a face detection algorithm, which allowed the humanoid robot to detect online the participants’ eyes and establish a real-time eye contact with them (for the output of the algorithm see Fig. 1, panel i). Using humanoid robots to examine human social cognition allows for excellent experimental control and, at the same time, ecological validity. Robots allow for manipulation of behavioral parameters in a controlled and modular way^34^, and can be programmed to behave contingently on the human behavior^35^. Embodied humanoid robots allow for a higher ecological validity relative to screen-based stimuli, as they increase social presence^35,36^. A humanoid robot compared to a virtual agent shares our environment and can make changes in the environment by, for example, manipulating objects. Humanoid robots can elicit the mechanisms of social cognition in a similar way as human-human interaction^35–37^. Moreover, eye contact with a robot increases its subjective social evaluation, attribution of intentionality and engagement (for a review see^35^).

**Figure 2:**
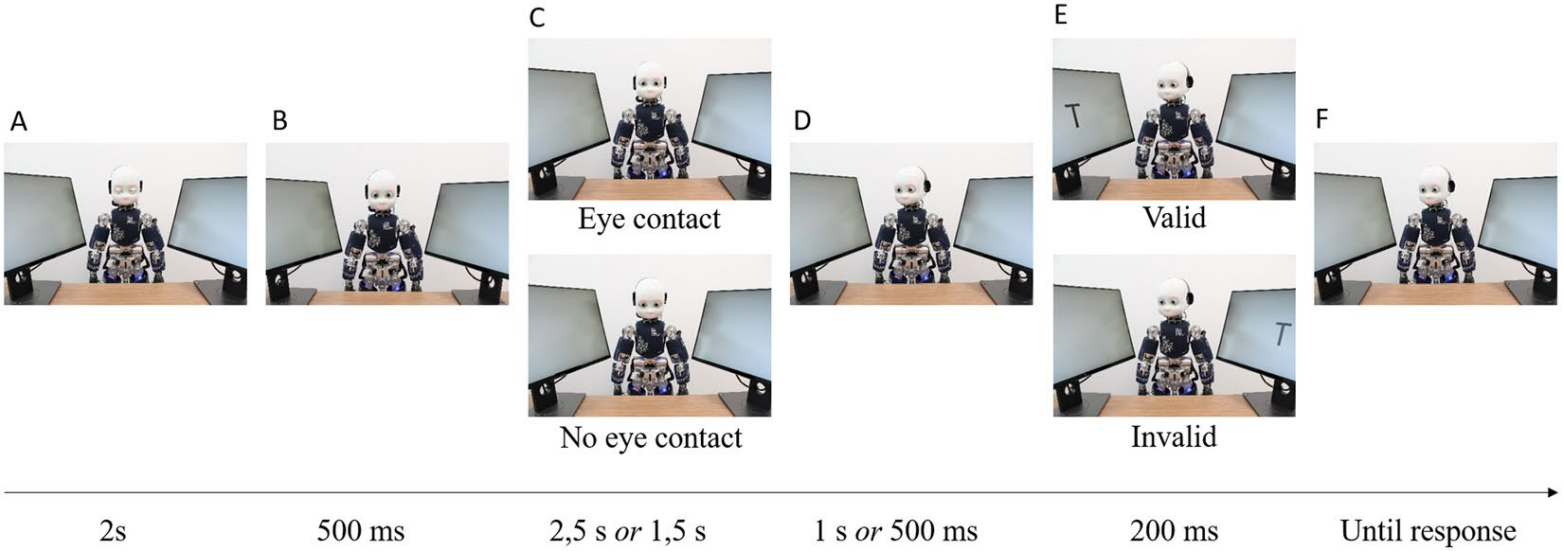
Experimental procedure. The robot (iCub) starts with its eyes closed for 2 s (**A**). Subsequently, it opens the eyes for 500 ms (without moving the head) (**B**). Then, iCub looks either down (no eye contact) or towards the participants’ eyes (eye contact) for 2.5 s (Experiment 1) or 1.5 s (Experiment 2) (**C**). After this, iCub moves its head laterally to gaze towards a potential target location (**D**). After 1 s (Experiment 1) or 500 ms (Experiment 2), the letter V or T appears randomly on one of the screens for 200 ms (**E**). The participant (not shown) identifies the target by pressing the mouse button (left or right) (**F**).

The aim of our study was to examine whether real-time eye contact modulates the GCE depending on the validity of the cue. In two experiments, we employed a gaze/head cueing paradigm, where the iCub^38^ was positioned between two lateral screens (Fig. 1, panel ii), on which targets were presented. In one condition, iCub established eye contact and then gazed to one of the lateral locations (eye contact condition), while in the other condition, the robot looked down without establishing eye contact (no eye contact condition, see Fig. 2). The eye contact was manipulated across blocks. In order to check if eye contact differently engaged participants in the task, at the end of each block, participants were requested to answer the following question: “How much did you feel engaged with the robot?”. In Experiment 1 cue-target validity was 50% and the stimulus-onset-asynchrony (SOA) was 1000 ms. We hypothesized that given the pivotal role of eye contact in social interaction^16^, and previous findings supporting a larger GCE in direct gaze compared to non-direct gaze^21,26^, the eye contact might act as a source of top-down enhancement of the bottom-up reflexive component, in line with the dual-component of gaze-related attentional orienting^11^. Therefore, we expected a larger GCE in eye contact, compared to the no eye contact condition. Experiment 2 aimed at examining whether the social top-down component, exerted by eye contact, would interact with the other top-down component, namely the strategic one, which might also modulate reflexive mechanism of attentional orienting in response to directional gaze cues. To achieve this, we designed a task in which cue-target validity was counter-predictive (25%), and SOA was reduced to 500 ms. By using counter-predictive cues, we made sure that *strategically* it would be beneficial to avoid orienting attention towards the direction of the gaze^4,11^. In addition, we reduced the SOA to make little time available for top-down control over reflexive processes. We hypothesized that under these experimental conditions, any gaze cueing effect that would potentially be observed would be due to a reflexive component of attentional orienting^3,4^. On the other hand, lack of gaze cueing effects would suggest that top-down control penetrated the reflexive mechanism. The question of interest was whether the postulated top-down component related to social signal of the mutual gaze would be powerful enough to have an impact on the reflexive component, even when little time is allowed. That is, whether the top-down component would reduce (or eliminate) the gaze cueing effects resulting from the reflexive mechanism, as following the gaze of the robot under 25% validity would not be not an efficient strategy.

## Results

### Experiment 1

One participant with a number of errors exceeding 3 standard deviations (SD) from the overall mean (3.84% ± 3.52) was excluded from further analyses. Error trials (3.44%), RTs slower than 2000 ms, or 2.5 SDs above or below an individual’s mean for each condition were removed (2.2% of remaining trials). The mean number of the trials after removing the outliers was similar across conditions and equal to 37.75 ± 1.54 trials on average. For each participant, we computed the GCE as the difference in RTs between invalid and valid trials for the eye contact and the no eye condition separately. A positive GCE means that participants responded faster to validly- compared to invalidly-cued targets, indicating that participants oriented their attention to the location gazed-at by the robot. A negative value of the GCE reflects, on the other hand, faster responses to invalidly- compared to validly-cued targets, suggesting that participants oriented their attention to the opposite direction than that of iCub’s gaze. GCEs were submitted to a repeated-measures analysis of variance (ANOVA) with gaze type (eye contact, no eye contact) as within-participants factor. Furthermore, one-sample t-tests were applied in order to calculate if the average GCE in both condition statistically differed from a normal distribution with a zero mean. Since the validity was randomized across the entire experiment and thus it was not constant in each block, an additional analysis was conducted according to validity rate per block. More specifically, a linear regression was run to investigate if GCE magnitude was predicted by the rate of validity of the block. For this analysis, the GCE was computed for each participant in each block. Then, the blocks were categorized according to the validity rate into three categories: low (valid trials < 50%), middle (valid trials = 50%) and high (valid trials > 50%). Furthermore, mean ratings for social engagement were analyzed using a Wilcoxon signed-rank test in order to compute the statistical difference between eye contact vs. no eye contact blocks. Spearman’s rank-order correlation coefficient was computed to assess the relationship between GCE and ratings of engagement.

#### Gaze cueing effect

**Figure 3:**
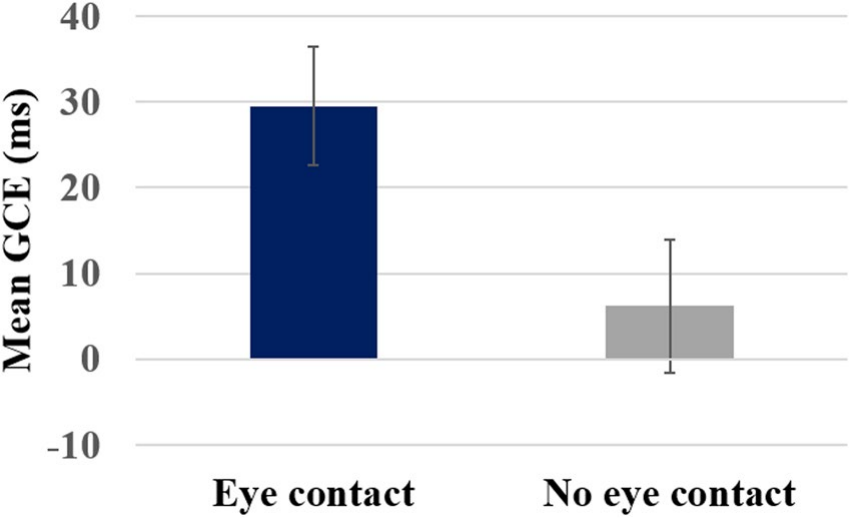
GCE (ms) as a function of gaze condition (eye contact vs. no eye contact). Error bars represent standard error of the means.

The analysis revealed a significant main effect of gaze type, F (1, 32) = 7.38, p = 0.01, η_p_² = 0.19 indicating a larger GCE for the eye contact (M_eye contact_ = 29.5, SEM = 7.02) compared to the no eye contact condition (M_no eye contact_ = 6.17, SEM = 7.8). One-sample t-tests showed that GCE in eye contact condition was statistically larger than 0, t (32) = 4.2, p < 0.001, 95% CI [15.2, 43.8] while the GCE in no eye contact condition did not significantly differ from 0, t (32) < 1, 95% CI [−9.8, 22.14], see Fig. 3. The multiple regression analysis indicated that gaze condition and validity rate significantly predicted the GCE magnitude, F (2,195) = 11,071, p < 0.001, R² = 0.102. However, only gaze condition (eye contact vs. no eye contact) added significantly to the prediction, β = −40.46, t (195) = −4.57, p < 0.001.

#### Error analysis

A paired sample t-test showed that the percentage of error trials did not significantly differ between the eye contact and no eye contact conditions, t(32) = 1.1, p = 0.29 (M_eye contact_ = 3.67%, SEM_eye contact_ = 0.59, M_no eye contact_ = 3.22%, SEM_no eye contact_ = 0.43). The percentage of error trials in valid condition was subtracted from the percentage of error trials in invalid condition for both gaze conditions. A paired sample t-test showed that the percentage of error trials did not significantly differ between the eye contact and no eye contact conditions, t(32) < 1, p = 0.42 (M_eye contact_ = 0.68%, SEM_eye contact_ = 0.79, M_no eye contact_ = 1.44%, SEM_no eye contact_ = 0.57).

#### Engagement rating

**Figure 4:**
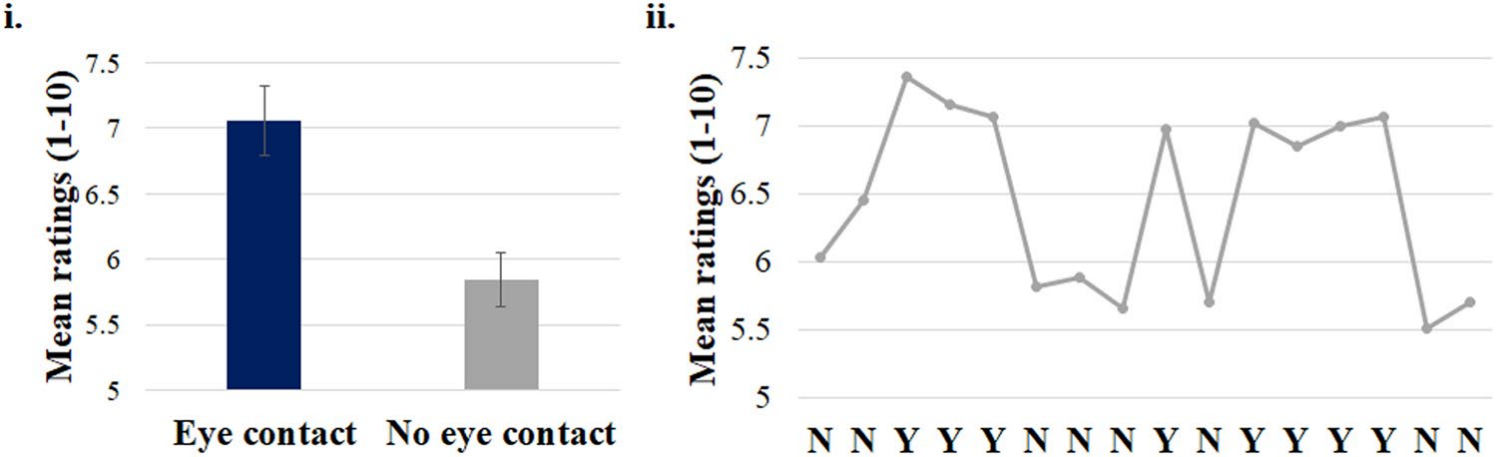
Panel i.: Average engagement ratings across conditions (eye contact, no eye contact). Panel ii: Mean engagement ratings across blocks (Y = eye contact; N = no eye contact). Error bars represent standard error of the means.

Participants rated the eye contact condition as more engaging than no eye contact, Z = −4.54, p < 0.001 (M_eye contact_ = 7.10, SEM = 0.27, M_no eye contact_ = 5.84, SEM = 0.21). The mean ratings of each gaze condition overall and across blocks are presented in Fig. 4. No correlation between the engagement ratings and the mean GCE emerged both for the eye contact, *r* = 0.07, n = 33, p = 0.70, and the no eye contact condition, *r* = 0.21, n = 33, p = 0.23.

### Discussion

Experiment 1 examined the impact of eye contact on orienting of attention driven by non-predictive gaze cues. GCE occurred in the eye contact condition, but not when there was no eye contact. Validity rate did not predict GCE magnitude, suggesting that the GCE was not related to the short-term variations in cue predicitivity, when the task was overall non-predictive (50% validity). Participants rated the eye contact condition more engaging, as compared to the condition with no eye contact. This is also reflected in the engagement ratings in each block, where participants repeatedly rated higher the blocks with eye contact (see Fig. 4, panel ii), as compared to the no eye contact condition.

Interestingly for the purposes of this paper, our results showed no significant GCE in the condition with no eye contact. It was a striking result, given that the directional cue of the robot’s head movement was a very salient signal. Therefore, a reflexive component should have also been present in the condition with no eye contact, in line with the idea of dual-component of attentional orienting in gaze cueing^11^ and the dual-model of spatial orienting of attention^39^. In line with these accounts, the reflexive component is a fast-acting mechanism with a transient facilitatory period, elicited by salient signals. A voluntary orienting component emerges slower and has a sustaining effect of attention orienting towards cued locations^5,39^. In Experiment 1, the SOA of 1000 ms might have caused the reflexive component to fade away. We set out to examine the more reflexive component of gaze-related attentional orienting in Experiment 2; and to address the question if eye contact would have an impact on the reflexive orienting of attention.

### Experiment 2

One participant with a number of errors exceeding 3 standard deviations (SD) from the overall mean (4.68% ± 3.35) was excluded from further analyses. Error trials (4.2%), RTs slower than 2000 ms, or 2.5 SDs above- or below an individual’s mean for each condition were removed (2.4% of all remaining trials). The mean number of the trials after removing the outliers was: 119.7 ± 3.15 for the eye contact and 119.5 ± 3.64 for the no eye contact condition. The average percentage of valid and invalid trials was similar across gaze conditions and equal to: M = 23.45 ± 0.99 (%) for valid trials and M = 70 ± 2.05 (%) for invalid trials.

The GCE was computed as in Experiment 1. In order to evaluate the effect of eye contact, the GCE was submitted to a repeated measures ANOVA with gaze type (eye contact, no eye contact) as within-participants factor. In addition, one-sample t-tests were conducted in order to calculate if the average GCE in both condition statistically differed from a normal distribution with a zero mean. Mean ratings for social engagement were analyzed using a Wilcoxon signed-rank test in order to compute the statistical difference between eye contact vs. no eye contact blocks. Finally, Spearman’s rank-order correlation coefficient was computed to assess the relationship between GCE and ratings of engagement.

#### Gaze cueing effect

**Figure 5:**
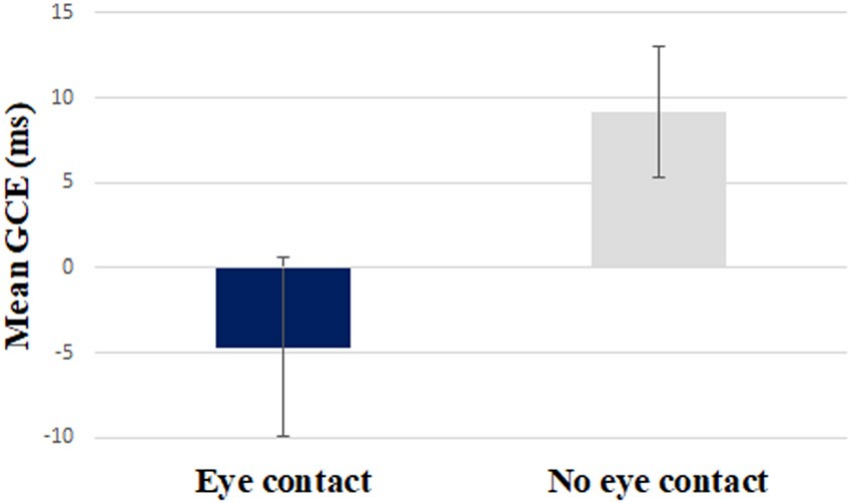
GCEs (ms) as a function of gaze condition (eye contact, no eye contact). Error bars represent standard error of the means.

The analysis revealed a significant main effect, F (1, 32) = 4.87, p = 0.035, η_p_² = 0.13, indicating a larger GCE in the no eye contact (M_no eye contact_ = 9.16, SEM = 3.9) compared to the eye contact condition (M_eye contact_ = −4.69, SEM = 5.25). One-sample t-test showed that only in the no eye contact condition the GCE was significantly different from 0, t(32) = 2.33, p = 0.03, 95% CI [1.15, 17.17], while the GCE in the eye contact condition did not significantly differ from 0, t(32) < 1, 95% CI [−15.38, 6.0], see Fig. 5.

In order to check if the results of Experiment 2 were not affected by the lower number of valid trials (25%) compared to invalid (75%), we conducted an additional analysis on a randomly selected subset of invalid trials (randperm function in Matlab). We repeated the main analysis on such a subset of trials. We computed the GCE, in a similar fashion as for the main analysis, and we submitted GCE to a repeated-measures ANOVA with gaze type (eye contact, no eye contact) as within-participants factor. The analysis reveal a stable pattern of results as indicated by the significant main effect, F (1, 32) = 7.1, p = 0.012, ηp² = 0.18, indicating a larger GCE in the no eye contact (M = 11.92, SEM = 4.3) compared to the eye contact condition (M = −8.9, SEM = 6.23). Moreover, in line with the results of the main analysis, one-sample t-test showed that only in the no eye contact condition the GCE was significantly different from 0, t(32) = 2.77, p = 0.01, 95% CI [3.16, 20.7], while the GCE in the eye contact condition did not differ significantly from 0, t(32) = −1.43, p = 0.16, 95% CI [−21.6, 3.8]. Results of this additional analysis mirror the pattern of the main analysis.

#### Error analysis

A paired sample t-test showed that the percentage of error trials did not significantly differ between the eye contact and no eye contact conditions, t(32) < 1, p = 0.78 (M_eye contact_ = 4.3%, SEM_eye contact_ = 0.42, M_no eye contact_ = 4.2%, SEM_no eye contact_ = 0.52). Similar to Experiment 1, the percentage of error trials in valid condition was subtracted from the percentage of error trials in invalid condition for both gaze conditions. A paired sample t-test showed that the percentage of error trials did not significantly differ between the eye contact and no eye contact conditions, t(32) < 1, p = 0.9 (M_eye contact_ = 0.22%, SEM_eye contact_ = 0.58, M_no eye contact_ = 0.32%, SEM_no eye contact_ = 0.56).

#### Engagement ratings

**Figure 6:**
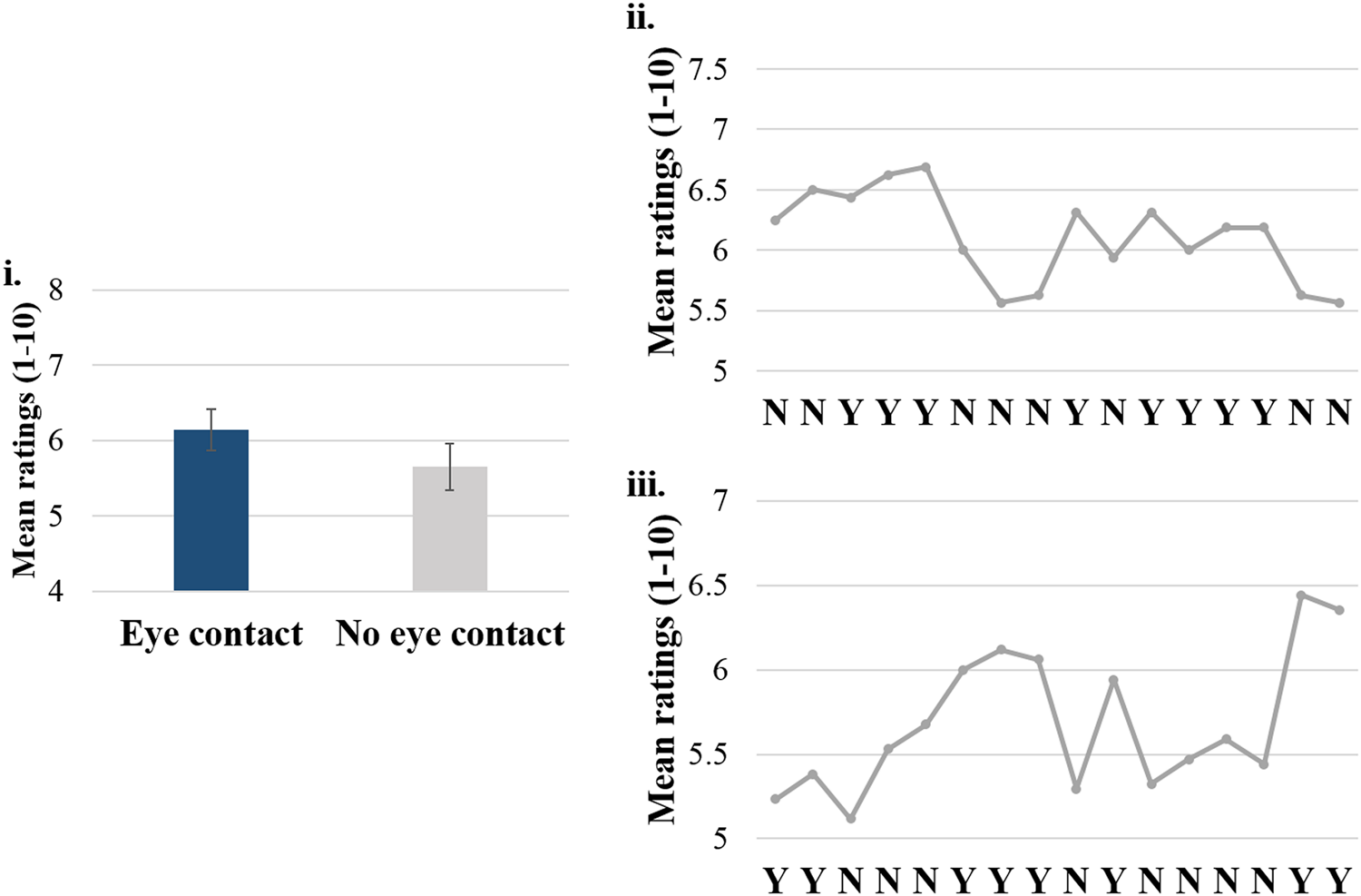
Panel i: Average engagement ratings averaged across conditions (eye contact, no eye contact). Panel ii & iii: Mean engagement ratings across block types (Y = eye contact; N = no eye contact) and the two block sequences that were counterbalanced across participants (panel ii: Sequence type *a*, panel iii: Sequence type *b*). Sequence type *a* starts with two blocks of no eye contact condition (N), while Sequence *b* starts with two blocks of eye contact condition (Y) and consists of the opposite gaze blocks compared to Sequence type *a*.

Overall, participants rated the eye contact condition as more engaging Z = −2.69, p = 0.007 (M_eye contact_ = 6.14, SEM = 0.28, M_no eye contact_ = 5.65, SEM = 0.31). The mean ratings for each gaze condition for the whole experiment and across blocks are presented in Fig. 6. There was no correlation between the rating scores and the mean GCE across participants for the eye contact condition, r = −0.22, n = 33, p = 0.22 and also for the no eye contact condition, r = 0.16, n = 33, p = 0.39.

### Discussion

Experiment 2 examined the effect of eye contact on the reflexive orienting of attention, that is, when following gaze cues is not strategically efficient for the task. To this end, we reduced the SOA from 1000 ms (Experiment 1) to 500 ms, and the gaze validity from 50% to 25% (i.e. the gaze cue was counter-predictive in the 75% of the trials). Results showed that a GCE statistically different from 0 was observed only in the no eye contact condition. Given the counter-predictive design of the task and the relatively short SOA, the observed GCE can be interpreted as being due to reflexive orienting of attention. This effect was not observed in the eye contact condition, suggesting an active top-down suppression in this case. Interestingly, despite the lack of GCE in the eye contact condition, participants rated the eye contact condition as more engaging then the no eye contact one.

## General Discussion

In the present study, we examined whether real-time eye contact influences GCE in a more ecologically valid scenario than classical screen-based paradigms. To this aim, we designed a gaze-cueing paradigm involving an embodied humanoid robot iCub. In Experiment 1 (non-predictive cueing procedure, 1000 ms SOA) we observed GCE for the eye contact condition, but not for the no eye contact condition. Experiment 2 (counter-predictive cueing procedure, 500 ms SOA) showed a reverse pattern. In both experiments, participants rated as more engaging the eye contact condition, compared to no eye contact condition.

Our results suggest that the GCE is a result of an interaction of a bottom-up reflexive orienting of attention, with top-down modulatory mechanisms related to strategic control and social engagement. In the case of non-predictive cues and relatively long SOA (Experiment 1), for the eye contact condition, the observed GCE might have been a combination of bottom-up mechanism and a top-down social enhancement, in line with previous literature^10–12^. This enhancement might have occurred because the eye contact condition was more engaging and/or rewarding, which was supported by the subjective ratings of engagement. Furthermore, it has been previously shown that eye contact positively modulates reward-related neural circuitry, as indicated by the activation of dopaminergic systems when pleasing faces are presented with a direct gaze compared to averted^40^. Similarly, Schilbach and colleagues^41^ showed that other contingent behaviors, such as initiating a contingent gaze sharing, can also activate reward-related brain regions (i.e., the ventral striatum). Since eye contact was more engaging, participants might have been more prone to follow the gaze of iCub when it engaged them in a more social context of eye contact.

In the no eye contact condition, no GCE was observed. This might have been due either to active suppression of the bottom-up reflexive component, or due to that the bottom-up component was not enhanced further by the social/engaging/rewarding context, and thereby it faded away with time. Although the present data cannot conclusively support one of the two interpretations, we speculate that it is more likely that the bottom-up mechanism simply faded away for the no eye contact condition, in line with literature showing that the bottom-up mechanisms of attention orienting are transient and short-lived^39^. This reasoning is further supported by another study^42^ in which GCE effects were found for both eye contact and no eye contact in non-predictive cueing procedure with 500 ms. In this case, it might be argued that the reflexive bottom-up mechanism was still observed (not yet faded away) due to 500 ms SOA.

One might argue that top-down active suppression of reflexive attentional orienting needed 1000 ms to develop, and hence it was observed in the present Experiment 1 but not in the other study with 500 ms SOA. However, Experiment 2 of the present study speaks against this interpretation, as in Experiment 2, in the eye contact condition, top-down suppression of reflexive component was already present at 500 ms SOA. Taken together, we argue that it is more likely that in Experiment 1, lack of GCE in no eye contact condition was due to temporal fading away of the reflexive component, rather than active suppression thereof.

On the other hand, in the case of counter-predictive cueing (Experiment 2), where GCE is most likely the signature of reflexive orienting of attention, we observed the reflexive mechanism in the no eye contact condition. Interestingly, for the eye contact condition, the GCE was not observed, suggesting top-down influence. Since in the counter-predictive cueing procedure, following the direction of gaze was very inefficient for the task (most of the times, following gaze direction led to focusing on the wrong location in terms of subsequent target appearance), it was strategically better to suppress orienting of attention in the direction of the gaze. Hence, due to a more engaging social signal in the eye contact condition, top-down control might have already been activated, while in the no eye contact condition, the reflexive component was still pronounced, resulting in significant GCE.

Taken together, our results suggest that when a socially rewarding/engaging signal is detected (as evidenced by engagement ratings), strategic top-down control might be more likely to be activated – which either enhances or suppresses activation of the attentional network, dependent on predictivity of the cue, and the best strategy to efficiently solve the task. When following the gaze is strategically equally sensible as not following the gaze, which is the case of our Experiment 1 (50% validity), the reflexive component of attentional orienting might be enhanced due to socially engaging eye contact. This allows the attention-related activity to be larger and/or last longer than the default reflexive component. This is in line with the idea that the top-down mechanisms of attentional orienting have a longer-lasting effect than the transient, reflexive component^4,5,39^. On the other hand, when following the gaze would be inefficient, and thus strategically would not make sense, as in Experiment 2 (25% validity), the engaging condition of eye contact presumably induces active suppression of the reflexive component of attentional orienting. Indeed, when a context is more engaging or socially rewarding (as in the case of our eye contact condition), top-down control can be potent enough to suppress the reflexive component of attentional orienting in response to directional gaze. However, in the case of no (socially) rewarding/engaging signal (i.e. no eye contact), the strategic top-down control might be less likely to be activated. Therefore, the default reflexive attentional orienting mechanism, related to gaze direction might be more prominent. This mechanism enhances processing of the target at the cued, relative to uncued, location, but the enhancement – being bottom-up – is likely transient^39^. Therefore, GCE are observed for a short SOA (500 ms), both for non-predictive^42^ and counter-predictive cues (Experiment 2). However, this enhancement fades away in cases where SOA is longer (1000 ms, Experiment 1).

In sum, results of the present study showed that using more interactive protocols with embodied presence of a humanoid robot allow for more ecological validity whilst maintaining experimental control. Such approach provides novel insights into the mechanisms of social cognition. In the case of our study, we showed that social signals such as gaze contact have an impact on the reflexive mechanism of gaze-related orienting of attention through activation of top-down strategic control processes.

As a final remark, we highlight the importance of the dissociation that we observed between subjective reports of engagement and the GCE. This is of relevance not only for social cognitive neuroscience and experimental psychology but mainly for the research field of human-robot interaction (HRI). In this field, most of studies rely on subjective reports. However, our results showed that self-reports do not reveal all the information about the underlying cognitive mechanisms. More specifically, we showed that the impact of eye contact on engagement ratings was similar, independently of cue predictivity. That is, eye contact always elicited higher engagement ratings, as compared to no eye contact. Interestingly, the GCE did not follow the same pattern, indicating a dissociation between subjective ratings and the objective measure of social engagement (i.e. the GCE), which is in line with previous findings of Martini *et al*.^7^. These findings suggest that in order to target the entire spectrum of cognitive mechanisms involved in HRI (or any other social interaction), one needs to supplement subjective reports with objective measures.

## Methods

### Participants

The sample size was estimated via a priori power analysis using G*Power^43^. The analysis yielded a sufficient number of 30 participants, adopting the effect size of a similar previous study^44^: dz = 0.53, α = 0.05, and 1-β = 0.80. In total, thirty-four healthy participants (mean age = 26.74 ± 6.45, 4 left handed, 17 female) took part in the Experiment 1 and thirty-four new participants (mean age = 26.18 ± 4.03, 5 left handed, 19 female) took part in Experiment 2. Participants received honorarium (15 €) for their participation. All had normal or corrected-to normal vision, and were debriefed about the purpose of the study at the end of the experiment. Both Experiment 1 and Experiment 2 were conducted in accordance with the ethical standards laid down in the 2013 Declaration of Helsinki and were approved by the local ethical committee (Comitato Etico Regione Liguria). The experiments were performed at the Istituto Italiano di Tecnologia. All participants provided written informed consent prior to participation. Data were stored and analyzed anonymously. The data related to this study can be accessed online at 10.5281/zenodo.1492607.

### Stimuli and Apparatus

The apparatus and stimuli were constant across experiments. The experiments were carried out in an isolated and noise-attenuated room. Participants were seated face-to-face with the iCub robot placed at the opposite side of the desk at a distance of 125 cm. iCub was mounted on a supporting frame and its eyes were aligned with participants’ eyes at 124 cm from the floor. iCub’s gaze shifts were always embedded in a head movement, in order to make them more naturalistic. The gaze could be directed (together with the head movement) to five different positions: “resting” - towards a location in space between the desk and participants’ upper body, “eye contact” - towards participants’ eyes (based on the output of face extraction algorithm, see subsection “iCub and algorithms”), “no eye contact” - towards the table, “left” - towards the location of the target on the left screen, and “right” - towards the location of the target on the right screen (see Table 1 for the x, y, and z coordinates of the robot gaze from the robot frame of reference, i.e., robot’s waist). The z-coordinate of “resting” and “no eye contact” positions were calculated starting from z-coordinate of “eye contact” gaze, in order to maintain the z-value for the resting condition equally distanced from the z-value of “eye contact” and “no eye contact” conditions (see Table 1). Importantly, the height of robot’s gaze prior to directional shift was equally distanced from the “left”/“right” position for both eye contact and the condition with no eye contact. Similarly, the amplitude of the gaze shift on the horizontal axis (y coordinate) was equal for left- and right- directed gaze shift (see Table 1). These coordinates were predetermined in order to ensure that the distance required to reach the end point (left or right) was the same both for the eye contact and for condition with no eye contact. Two screens (21.5 inches) were used for stimuli presentation and were situated laterally on a desk at a viewing distance of 105 cm from the participant’s nose apex, see Fig. 1. The screens were both tilted back approximately by 12° from the vertical position and were rotated by 76° to the right or left, to create the impression that iCub can “see” the stimuli presented on the screens. The screens were positioned 75 cm apart (center-to-center) and the stimuli were letters V or T (3°32′ high, 4°5′ wide). iCub, stimulus presentation, and data collection were controlled by an experiment programmed in C++ using the Ubuntu 12.04 LTS operating system.

**Table 1:** Positions of robot gaze from robot frame of reference (in m).

Positions of robot gaze	x	y	z
Resting	−0.78	0.0	0.16
No eye contact	−0.78	0.0	0.04
Left	−0.78	−0.35	0.16
Right	−0.78	0.35	0.16
Eye contact	−0.78	0.0	0.28

#### iCub and algorithms

iCub is a humanoid robot (size: 104 × 34 cm), with 3 degrees of freedom in the eyes (common tilt, vergence, and version) and three additional degrees of freedom in the neck (roll, pitch, yaw). YARP (Yet Another Robot Platform) is used as the iCub middleware^45^. YARP is a multi-platform open-source framework, which comprises a set of libraries, protocols, and tools, supporting modularity and interoperability. To control the eyes and the neck of iCub, we used the YARP Gaze Interface, iKinGazeCtrl, from the available open source repository (https://github.com/robotology/iCub-main/tree/master/src/modules/iKinGazeCtrl), which allows the control of iCub’s gaze through independent movement of the neck and eyes following a minimum-jerk velocity profile^46^. In our gaze cueing procedure, iCub moved its entire head to one of the sides, not only its eyes, to make its behavior more naturalistic (see Supplementary Material). The vergence of the eyes was set to 5 degrees and maintained constant. The vergence was locked because the combined movement of neck and eyes using the iKinGazeCtrl controller produces an overshooting in the position of the eyes which would result in a very unnatural cueing procedure (see Roncone *et al*.^46^ for a qualitative comparison of the velocity profiles between typical gaze shifts in humans and iCub’s using iKinGazeCtrl). Additionally, previous studies have reported similar attentional effects produced by head and gaze cueing^47,48^, thereby encouraging us to use the entire head movement of the iCub. The trajectory time for the movement of eyes and neck was set for this experiment to 200 ms and 400 ms respectively, to maintain the impression of a smooth and naturalistic movement. The human eyes were detected using the face detector of the (https://github.com/robotology/human-sensing) repository, which uses the dlib library (http://dlib.net), see Fig. 1- panel i. Informed consent for publication of Fig. 1-panel i was obtained.

### Procedure

In both experiments, participants were instructed to keep their eyes fixated on the face of the robot and to not move their eyes towards the screens. The latter requirement was also the best possible strategy for the task, as the letters on the screen were presented in peripheral vision, so moving the eyes toward one screen would mean missing the target, if it appeared on the opposite one. The experimenter monitored online eye movements of participants through the iCub cameras in order to check that at the beginning of each trial they were following the instructions and fixated at the robot’s face. Participants were asked to hold a mouse with their thumbs placed on the buttons and to identify the target as fast and as accurate as possible. Half of the participants pressed the left key for the V stimulus and the right key for the T stimulus (stimulus-response mapping 1). The other half was assigned the opposite stimulus–response mapping (mapping 2). At the end of each block, participants were requested to answer aloud the following question: “How much did you feel engaged with the robot (1–10)?”. The answer was noted down by the experimenter and the participant continued to the next block by pressing the middle mouse button.

#### Experiment 1

A full experimental session lasted about 25 minutes. The duration of all events include the robot movement which lasted for 400 ms, equivalent to the neck trajectory time. The sequence of events (cf. Fig. 2) was the following: Each trial started with the robot having its eyes closed at the resting position. After 2 s, the robot opened its eyes for 500 ms. During this time, the robot extracted information related to the position of the face and the eyes of the participant without making any movement. Then, it looked either to the predefined position: down, for the condition with no eye contact, or direct to the eyes of a participant in the eye contact condition. The whole duration of this phase was 2,5 s (actual eye contact duration: ~2 s). Subsequently, the robot’s head and eyes shifted to either the left or the right screen. Head direction was uninformative with respect to target location (i.e. cue-target validity = 50%). Following the onset of the robot’s gaze shift, after 1 s, a letter appeared on one of the lateral screens for 200 ms. After 200 ms, the screens turned blank until the participants responded. Target duration was defined following the gaze-cueing procedure with iCub applied in Wykowska *et al*.’ study^42,44^. Experiment 1 consisted of 160 pseudo-randomized trials, divided into 16 blocks of 10 trials each. The blocks were randomly assigned to one of the gaze condition: eye contact or no eye contact. The order of blocks was constant across participants. Cue-target validity was randomized across trials, both for eye contact and no eye contact conditions, throughout the experiment.

#### Experiment 2

A full experimental session lasted 40 minutes. The procedure was the same as in Experiment 1 with only three exceptions. First, a 75% ratio of invalid trials were included in each block, in line with the counter-predictive nature of the cueing procedure. Second, we reduced the SOA from 1000 ms to 500 ms to address the more reflexive component of gaze-related attentional orienting^3^. It is important to note here that the SOA in a naturalistic scenario with an entire head movement is not comparable to classical gaze cueing paradigms where there is no gradual transition of the gaze shift. Therefore, what seems to be a relatively long SOA in classical paradigms (500 ms) appears much shorter when the entire head movement is displayed, given that the SOA is counted from the onset of the movement to its final position. Finally, in order to compensate for the shorter SOA (half the duration of the SOA in Experiment 1), the whole phase of gaze manipulation (including eye contact/gaze down) was also reduced to 1.5 (actual eye contact duration: ~1 s) so that the ratio of duration between eye contact/no eye contact and SOA would remain similar. Indeed, several studies showed that long time of direct gaze is an ostensive signal^5,49,50^. This combined with the counter-predictive nature of the task might have led to the robot have been perceived as aggressive or competitive, therefore yielding to a completely different social context compared with Experiment 1. In total, 256 pseudo-randomized trials were presented, divided into 16 blocks of 16 trials each. The order of the blocks was counter-balanced across participants, using either the same randomized sequence of Experiment 1 (Sequence type *a*) or the opposite (Sequence type *b*). We counterbalanced the Sequence of Eye contact/No-eye contact blocks in order to control for any potential effect of block order. Moreover, given the counter-predictive nature of the task in Experiment 2 we wanted to ensure that the strategical top-down component was not affected by the condition of the first block (i.e. eye contact or no-eye contact). An analysis on GCE as a function of block sequence showed that block sequence did not affect the GCE (all Fs < 1), thus it was not included in the in our analyses as a factor.

## Electronic supplementary material

Supplementary material
